# Mental health disorders among post graduate residents in Kenya during the COVID-19 pandemic

**DOI:** 10.1371/journal.pone.0266570

**Published:** 2022-04-04

**Authors:** Sayed K. Ali, Jasmit Shah, Katie Du, Nidhi Leekha, Zohray Talib

**Affiliations:** 1 Department of Medicine, Aga Khan University Hospital, Nairobi, Kenya; 2 Brain and Mind Institute, Aga Khan University, Nairobi, Kenya; 3 Department of Radiology, Aga Khan University Hospital, Nairobi, Kenya; 4 Department of Medical Education, California University of Science and Medicine, Colton, CA, United States of America; New York City Department of Health and Mental Hygiene, UNITED STATES

## Abstract

**Background:**

Healthcare workers, including residents, are prone to various mental health disorders especially given the context of the COVID-19 pandemic. Residents, particularly, are already under undue stress due to their respective training program demands.

**Methods:**

This cross-sectional, online survey-based study from August to November 2020 collected demographic and mental health measurements from all residents at the Aga Khan University Hospital, Nairobi. The questionnaire investigated demographic variables, information regarding direct care of COVID-19 patients, prior history of mental health and mental health outcomes using the Patient Health Questionnaire, Generalized Anxiety Disorder scale, Insomnia Severity Index, Impact of Event Scale–Revised Questionnaire and Stanford Professional Fulfillment Index Questionnaire.

**Results:**

A total of 100 residents completed the survey (participation rate 77.5%). Participants were about equal in gender (women [53%]), with a median age of 31.28 years, and majority were single (66.7%). A total of 66 participants (66%) were directly engaged in COVID-19 care. Depression: 64.3%, anxiety: 51.5%, insomnia: 40.5%, distress: 35.4%, and burnout: 51.0% were reported in all participants. Statistical significance was found in median depression, professional fulfillment and interpersonal disengagement when comparing frontline resident directly involved in care of COVID-19 patient versus second line residents.

**Conclusion:**

Residents directly involved with caring for COVID-19 patients had statistically higher incidences of depression and interpersonal disengagement and lower professional fulfillment compared to second line residents. Keeping in mind the limited resources in sub-Saharan Africa, urgent and geographically specific strategies are needed to help combat mental health disorders in this specific population.

## Introduction

Since the first confirmed COVID-19 case in Africa in early 2020, there has been over 8 million cases across the continent with over 320,000 cases and about 5,500 deaths in Kenya as of January 2022 [1, 2]. Prior to the first confirmed COVID-19 case in Kenya, the government had already began drafting measures to limit physical contact and virus transmission [3]. The social consequences of these restrictions, not exclusive to Kenyan society, in combination with the unpredictable and uncertain nature of both the seriousness of the disease as well as governments’ planned courses of action has greatly contributed to the increased psychiatric morbidity worldwide [4].

Moreover, studies have found both frontline (directly involved in the care of COVID-19 patients) and second line (not directly involved in the care COVID-19 patients) healthcare workers (HCWs) are especially at-risk for adverse mental health outcomes such as increased levels of anxiety, depression and insomnia [5, 6]. Mental health assessment results of medical residents mirrored those of other types of HCWs, suggesting that psychological distress is not limited to specific subsets of the HCWs population or professional hierarchy [6]. Many studies assessing the mental health of medical residents through the COVID-19 pandemic, however, were completed in developed countries outside of sub-Saharan Africa (SSA), such as the U.S., Canada and Belgium [7–9]. In developing countries such as Kenya where a significant proportion of the population lives in poverty, high-density areas and suffers from co-existing diseases or lacks access to adequate nutrition, the existing strain on the system may amplify pandemic-related healthcare delivery challenges. For example, while in the U.S. there were approximately 2.6 physicians per 1,000 people in 2014, in Kenya there was approximately 0.2 physician per 1,000 people [10]. Therefore, universal pandemic-imposed healthcare workplace stressors, such as concerns over sufficient supplies of personal protective equipment, testing kits, efficacy of treatment plans, exposure to the virus and heightened workloads, may have a disproportionate impact on HCWs in Kenya where there is already a high disease burden and severe resource constraints [11, 12].

Furthermore, medical residents may be at a further disadvantage compared to senior staff during such a pandemic given their subordinate status as physicians-in-training and potentially limited opportunities to initiate workplace changes for mental health reasons [8]. Schwartz et al. surveyed internal medicine residents at a New York City Hospital and found that approximately 1 in 5 resident physicians had considered suicide or self-harm since the onset of the pandemic [13]. Khusid et al. distributed a survey to all United States urology programmes and based on the responses, concluded that perceived access to personal protective equipment as well as support by their respective residency programme were both inversely correlated with depression and anxiety mental health outcomes [14]. In addition to workplace stressors, medical residents must adapt to drastic changes in their education, such as modified clinical placements and a shift towards online learning [15]. Online course delivery may be especially challenging in Kenya due to resource constraints [16]. Even in relatively wealthier countries, the increased reliance on technology has highlighted accessibility issues [17]. For example, students who do not have ready access to digital infrastructure, those living with disabilities or experiencing financial challenges due to COVID-19 may not be well-equipped and supported to effectively engage with online learning, potentially leading to increased anxiety and stress. Moreover, Zingaretti et al. found that the majority of interviewed plastic and esthetic surgery residents in Italy did not believe that the tools, such as webinars, used for teaching during COVID-19 could completely substitute in-person training opportunities; however, the authors acknowledged that virtual tools may help students increase their confidence in their skillsets and be effective supplementary materials [18]. The vulnerable position of medical residents imposed by intersecting pandemic-related stressors and the paucity of data from SSA highlights the importance of further investigating the impact of COVID-19 on HCWs, especially medical residents in SSA. This study therefore sought to determine the prevalence of mental health symptoms among residents directly involved with COVID-19 care.

## Methods

A cross sectional survey study was performed between August and November 2020 among all post graduate residents currently enrolled in nine different Graduate Medical Education (GME) programs at the Aga Khan University Hospital, Nairobi (AKUHN). Email addresses of all post graduate residents were obtained by the principal investigator from each department. Email invitations with a link to a voluntary, de-identified survey were sent to all 129 residents and all responses remained anonymous. In Kenya, the first wave peaked in early August 2020, whereas the second wave peaked around mid-November 2020. The online survey data was collected through Research Electronic Data Capture—REDCap (web interface of a research electronic data capture instance, powered by Vanderbilt and supported in part by the National Institute of Health) [19]. Online electronic consent was obtained from all the participants and they were allowed to withdraw from the survey at any time without any consequences. The survey was anonymous, and confidentiality of information was assured. Approval for this study was obtained from the Institutional Ethics and Review Committee AKHUN (Ref: 2020/IERC-95 (v2) and National Commission for Science and Technology & Innovation (NACOSTI/P/20/6257).

### Data collection

The survey questionnaire, in English, consisted of demographic characteristics, information regarding care of COVID-19 and mental health disorders. We defined “frontline” HCWs as those HCWs who reported in providing direct care (diagnosing, treating or providing nursing care) to COVID-19 patients whereas “second line” as not involved in care of COVID-19 patients Mental health disorders were measured using validated questionnaires: 9-item Patient Health Questionnaire (PHQ-9) (for depression) [20], 7-item Generalized Anxiety Disorder Questionnaire (GAD-7) (for anxiety) [21], 7-item Insomnia Severity Index Questionnaire (ISI) (for insomnia) [22], 22-item Impact of Event Scale–Revised (IES-R) (for distress) [23] and 16-item Stanford Professional Fulfillment Index Questionnaire (SPFI) (for burnout) [24].

The outcomes of these standardized questionnaires were interpreted as follows: Depression: normal (0–4), mild (5–9), moderate (10–14), and severe (15–27); Anxiety: normal (0–4), mild (5–9), moderate (10–14), and severe (15–21); Insomnia: normal (0–7), subthreshold (8–14), moderate (15–21), and severe (22–28); IES-R, normal (0–8), mild (9–25), moderate (26–43), and severe (44–88) distress and SPFI, burnout > 1.33 and professional fulfillment >3. The cutoff score for detecting symptoms of major depression, anxiety, insomnia, and distress were 10, 7, 15, and 26, respectively [25]. If the scores indicated a mental health issue, the participants were automatically notified after completion of the survey and directed to either a healthcare provider of choice or a mental health hotline provided by the hospital. A 24 hour 7-day hospital hotline was available for participants to access mental health services if required. In addition, all participants had the option to call the primary researchers if they had any questions or concerns about access to mental health services.

### Statistical analysis

Independent variables consisted of age, gender, marital status, ethnicity, prior history of mental health disorders and information regarding care of COVID-19 such as if frontline, how many COVID-19 patients care, enough resources to care for COVID-19 and adequately trained to treat COVID-19 patients. The dependent variables were all the mental health disorders such as depression, anxiety, insomnia, distress and burnout. Data was presented as frequencies and percentages or as medians with interquartile ranges (IQR). Kruskal-Wallis test and Fishers exact test was utilized to make comparisons between two groups. Data was analysed using SPSS version 20 and a p value of <0.05 was considered statistically significant.

## Results

A total of 100 residents completed the online survey (response rate = 77.5% (N = 129)). Participants were about equal in gender (women [53%%]), with a mean age of 31.28 years (IQR [29.96, 33.07]), and majority were single (66.7%). A total of 66 participants (66%) were directly engaged in COVID-19 patient care. Only 8.0% of the participants had prior history of any mental health disorder. Participant characteristics are reported in Table 1.

**Table 1:** Baseline characteristics of study participants (N = 100).

		(n = 100)
Age (n = 89) (median [IQR])		31.28 [29.96, 33.07]
Gender	Male	46 (46.0%)
	Female	53 (53.0%)
	Prefer not to disclose	1 (1.0%)
Marital Status (n = 99)	Single	66 (66.7%)
	Married	31 (31.3%)
	Other	2 (2.0%)
Ethnicity (n = 98)	African	81 (82.7%)
	Asian	14 (14.3%)
	Other	3 (3.1%)
Directly Cared with COVID	Yes	66 (66.0%)
	No	34 (34.0%)
Patients Cared (n = 66)	< 5 Patients	23 (34.8%)
	5–20 Patients	16 (24.2%)
	> 20 Patients	27 (40.9%)
Lost any Patients (n = 66)	Yes	35 (53.0%)
	No	31 (47.0%)
Enough Resources to care for COVID (n = 66)	Yes	50 (75.8%)
	No	16 (24.2%)
Adequately Trained for COVID	Yes	33 (50%)
	No	33 (50%)
Prior History of any mental health disorder	Yes	8 (8.0%)
	No	85 (85.0%)
	Not Sure	6 (6.0%)
	Prefer not to disclose	1 (1.0%)
Diagnosis	Anxiety	3 (37.5%)
	Depression	5 (62.5%)
Still have symptoms	Yes	3 (37.5%)
	No	5 (62.5%)

Depression, anxiety, insomnia, distress, and burnout were reported in 64.3%, 51.5%, 40.5%, 35.4%, and 51.0% of all participants. Statistical significance was found only in median depression scores, professional fulfillment and interpersonal disengagement when comparing frontline resident directly involved in care of COVID-19 patient versus those not involved in care of COVID-19 patients (eg, median depression scores among frontline vs second line: 6.50 [3.00, 11.00] vs 5.00 [3.00, 8.00]; p = 0.04, professional fulfillment among frontline vs second line: 3 [4.8%] vs 7 [21.2%]; p = 0.03), interpersonal disengagement among frontline vs second line: 29.2 [8.3, 50.0] vs 20.8 [0.0, 25.0] p < 0.01) (Table 2). The questionnaires demonstrated good internal consistency with a Cronbach alpha of 0.910, 0.938, 0.907, 0.976 and 0.932 for PHQ-9, GAD-7, ISI, IES-R and SPFI respectively.

**Table 2:** Baseline characteristics of study participants (N = 100).

			*Total*	*Have you ever directly cared for a patient with COVID-19*?		
				*Yes*	*No*	*P Value*
***PHQ-9***	** *Depression* **		** * * **	**(n = 64)**	**(n = 34)**	*0*.*29*
		None	35 (35.7%)	19 (29.7%)	16 (47.1%)	
		Mild	35 (35.7%)	23 (35.9%)	12 (35.3%)	
		Moderate	18 (18.4%)	14 (21.9%)	4 (11.8%)	
		Severe	10 (10.2%)	8 (12.5%)	2 (5.9%)	
		Score	6.0 [3.0, 11.0]	6.5 [3.0, 11.0]	5.0 [3.0, 8.0]	*0*.*04*
***GAD-7***	***Anxiety***			**(n = 63)**	**(n = 34)**	*0*.*46*
		Minimal	47 (48.5%)	27 (42.9%)	20 (58.8%)	
		Mild	33 (34.0%)	23 (36.5%)	10 (29.4%)	
		Moderate	6 (6.2%)	4 (6.3%)	2 (5.9%)	
		Severe	11 (11.3%)	9 (14.3%)	2 (5.9%)	
		Score	6.0 [3.0, 10.0]	6.0 [2.0, 8.0]	4.0 [1.0, 7.0]	*0*.*20*
***ISI***	***Insomnia***			**(n = 64)**	**(n = 34)**	*0*.*20*
		None	58 (59.2%)	33 (51.6%)	25 (73.5%)	
		Subthreshold	25 (25.5%)	19 (29.7%)	6 (17.6%)	
		Moderate	12 (12.2%)	9 (14.1%)	3 (8.8%)	
		Severe	3 (3.1%)	3 (4.7%)	0 (0.0%)	
		Score	6.0 [3.0, 11.0]	6.5 [3.0, 12.5]	5.00 [3.0, 8.0]	*0*.*23*
***IESR***	***Distress***		** **	**(n = 63)**	**(n = 33)**	*0*.*26*
		Normal	62 (64.6%)	39 (61.9%)	23 (69.7%)	
		Mild	13 (13.5%)	7 (11.1%)	6 (18.2%)	
		Moderate	3 (3.1%)	2 (3.2%)	1 (3.0%)	
		Severe	18 (18.8%)	15 (23.8%)	3 (9.1%)	
		Score	13.0 [2.0, 30.5]	14.0 [2.0, 38.0]	12.0 [0.0, 26.0]	*0*.*36*
		Avoidance	0.6 [0.1, 1.5]	0.8 [0.1, 2.0]	0.6 [0.0, 1.1]	*0*.*46*
		Intrusion	0.6 [0.0, 1.5]	0.6 [0.1, 1.6]	0.6 [0.0, 1.1]	*0*.*46*
		Hyperarousal	0.3 [0.0, 1.2]	0.3 [0.0, 1.5]	0.5 [0.0, 1.0]	*0*.*49*
***SPFI***	***Burnout***			**(n = 63)**	**(n = 33)**	*0*.*13*
		≤ 1.33	47 (49.0%)	27 (42.9%)	20 (60.6%)	
		> 1.33	49 (51.0%)	36 (57.1%)	13 (39.4%)	
	***Professional Fulfillment***		*** ***	**(n = 63)**	**(n = 33)**	*0*.*03*
		≤ 3.00	86 (89.6%)	60 (95.2%)	26 (78.8%)	
		> 3.00	10 (10.4%)	3 (4.8%)	7 (21.2%)	
	*Professional Fulfillment*		50.0 [31.3, 66.7]	50.0 [25.0, 62.5]	58.3 [50.0, 75.0]	*0*.*01*
	*Work Exhaustion*		50.0 [31.3, 68.8]	50.0 [31.3, 75.0]	37.5 [25.0, 56.3]	*0*.*06*
	*Interpersonal Disengagement*		25.0 [4.2, 41.7]	29.2 [8.3, 50.0]	20.8 [0.0, 25.0]	*<0*.*01*

## Discussion

A total of 100 residents enrolled in nine GME program at the AKUHN completed an online survey assessing demographic characteristics, information regarding care for COVID-19 patients and mental health parameters including depression, anxiety, insomnia, distress and burnout. More than half of those surveyed reported experiencing depression, anxiety and burnout. More than a third of those surveyed reported experiencing insomnia and distress. Pursing a residency can be a stressful time for most residents and this is certainly true during a pandemic when much more is expected from the residents. In fact, some studies have shown that being a resident is a predictive variable for psychological problems especially during pandemics such as COVID-19 [26].

In a study examining specifically the mental health of physician trainees in Saudi Arabia, Alsaywid et al. found that 42.2% of participants suffered from moderately severe to severe depression [27]. A similar study done in Qatar by Khoodoruth et al. reported high rates of depression, anxiety and stress among medical residents exposed to COVID-19 [28]. Furthermore, a study done in Brazil by Mendonça et al., looking at medical residents also found a high level of depressive symptoms specifically among second-year residents, while anxiety was more common among the fourth-year residents [29]. These results are consistent with our findings demonstrating high levels of depressive symptoms amongst residents. We also found a statistically significant difference in the median depression scores between residents working on the frontline and second line residents not involved in the care of COVID-19 patients. This result reinforces the findings of other studies that have demonstrated a correlation between degree of exposure to COVID-19 and adverse mental health outcomes [6]. Schwartz and colleagues looking at resident mental health at the epicenter of the COVID-19 pandemic in New York city found that more than 1 in 5 residents contemplated suicide of self-harm during the pandemic [13]. Comparatively, based on the PHQ-9 question 9 on suicidal thoughts, 13% of our residents’ sample reported having such thoughts. This is a concept that needs to be better explored in future studies especially in LMIC setting such as ours.

Collins et al. found that GAD-7 scores were significantly higher amongst surveyed senior surgical residents compared to interns [30]. As stated above, Mendonça et al. also found that anxiety was more common in fourth year residents during the COVID-19 pandemic [29]. Interestingly, a greater proportion of interns reported they were moderately or extremely anxious about possible COVID-19 infection compared to senior residents. This suggested to the authors that higher GAD-7 scores amongst surveyed senior surgical residents cannot at least entirely be attributed to anxiety over exposure but rather also owing to other stressors, such as uncertainty associated with transitioning from residency. These observations may help explain our finding of no statistical difference in GAD-7 anxiety scores between those of medical residents working on the frontline versus those of medical residents working on the second line. Frontline and second line residents may be affected by a myriad of similar stressors, such as increased workload and perceived impact of missing educational opportunities, that lead to similar GAD-7 scores despite differences in exposure to COVID-19.

Our study demonstrated a high burnout rate of 51% amongst residents consistent with what other authors found in their study examining burnout amongst medical residents during the COVID-19 pandemic [29, 31]. Lower rates of burnout were reported by psychiatry residents in a study conducted by Alkhamees et al. during the outbreak in Saudi-Arabia [32]. A study by Khalafallah et al. looking at the impact of the COVID-19 pandemic upon burnout among neurosurgery residents reported higher post-graduate years was associated with less burnout [33]. This trend is worrisome in especially younger HCWs in their early years of training who may not be as well adept and empowered to cope with various stressors during their training [8]. Unlike Civantos et al. who found a higher prevalence of burnout amongst female otolaryngology residents, fellows and attending physicians compared to their male counterparts, we did not see a statistically significant difference in burnout or other mental health outcomes between male and females residents involved in the care of COVID-19 patients [7]. Since the results of the questionnaires we used are dependent upon self-reporting, we may have not observed a difference in the mental health outcomes between male and female residents because men are less likely to report symptoms, leading to under diagnosis of mental health disorders [34]. Interestingly, Treluyer and Tourneux looking at burnout among pediatric residents during the outbreak in France, found no association between burnout and exposure to COVID-19 probably due to the low incidence of severe COVID-19 among children [35]. Due to the relatively low number of pediatrics residents present at our institution, we were not able to confirm this finding in our resident sample.

Alnofaiey et al. examined sleep disturbances amongst Saudi physicians during the pandemic and determined that almost 50% of residents showed some form of sleep disorders [36]. Similarly, Costa et al. found that 45% of their resident sample size reported poor sleep quality during the COVID-19 pandemic [37]. Similarly, San Martin et al. also reported that frontline healthcare workers in Spain developed more sleep disturbances with worsening quality of sleep during the outbreak [38]. Our study showed a high rate of insomnia amongst residents taking care of COVID-19 patients, but there was no significant difference in insomnia between first and second line residents. This is not surprising given the increased work hours and responsibilities of all residents especially during the pandemic.

Furthermore, 35.4% of our residents reported psychological distress, likely due to factors such as increased workload and probability of experiencing traumatic stress. Approximately 41% of residents directly involved with COVID-19 cared for 20 patients or more and over 50% of frontline residents reported losing patients through the pandemic. However, there was no significant difference in psychological distress between frontline and second line residents. Approximately 30% of primary care physicians in China reported high levels of distress related to the COVID-19 pandemic primarily due to low preparedness, impact of personal life and safety concerns related to COVID-19 [39]. Of note only 23% of oncology and radiation therapy residents and fellows surveyed in France reported experiencing psychological distress [40]. This lower prevalence rate may be due to better access to medical and mental health resources when compared to LMIC settings.

A study examining the knowledge, attitude and practices of Zambia medical laboratory professionals towards COVID-19 found that a majority of surveyed participants had good knowledge about COVID-19 [41]. Similarly, a cross-sectional analysis of 52 neurosurgery residents selected from various countries found that 60% reported satisfactory knowledge about COVID-19 [42]. Approximately half of our resident population reported inadequate training around COVID-19 shedding light on the need for additional education training primarily focusing on our resident population. Medical education in Africa in general is affected by the lack of formal pedagogical training for faculty, limited number of faculty to fulfill increasing demands, insufficient learning infrastructure available to students and minimal ingenuity towards designing novel teaching methods that better address the needs of medical students and residents in resource constrained settings [43].

Our study has several limitations. We did not have comparative studies on the depression, anxiety, insomnia, distress and burnout rates amongst this same group of participants from before the pandemic and thus it is difficult to discern if the pandemic worsened existing mental health disorders in residents. Even though our study looked at residents in one academic center, we believe our results mirror the increased mental health disorders in residents at other academic centers in and around Kenya. Despite our response rate being adequate, we also cannot exclude the possibility of a non-response bias where residents experiencing mental health disorders or residents coping well chose not to respond to the survey. Lastly, due to the low numbers of residents in each training program, we were unable to compare differences in mental health outcomes between residents in different training programs at our institution.

## Conclusion

The COVID-19 pandemic has been shown to adversely affect the mental health of HCWs around the world. Residents, already under undue stress due to pressures of their training program, are now under exceptional stress due to the pandemic. Our study, the first of its kind, sheds light on the increased mental health disorders in residents directly involved in COVID-19 patient care in Kenya. Furthermore, our study also highlights the need for urgent and specific mitigating strategies to help curb the increasing mental health burden faced by residents in SSA.

## Supporting information

S1 Data (XLSX)
